# Cefepime-Induced Seizures: The Overlooked Outpatient Adverse Reaction

**DOI:** 10.7759/cureus.9268

**Published:** 2020-07-19

**Authors:** Arshia Khorasani-zadeh, Indrit Greca, Kunal Gada

**Affiliations:** 1 Medicine, State University of New York (SUNY) Upstate Medical University, Syracuse, USA

**Keywords:** cefepime, seizure, toxicity

## Abstract

Cefepime is a 4th generation cephalosporin often used for its ability to cover gram-positives, gram negatives, anaerobic bacteria, and, most importantly, pseudomonas. Prior to initiation of cefepime, the medication is dosed based on the renal function to avoid a multitude of its toxicity profiles, including encephalopathy, aphasia, myoclonus, seizures, and nonconvulsive status epilepticus. These risks are increased in the presence of renal impairment. We present a case of a 65-year-old woman who had presented to the emergency department (ED) two weeks after initiation of outpatient IV cefepime therapy with concerns of altered mentation and decreased oral intake. In the ED, the patient was noted to have a creatinine: 5.77 (baseline of 0.76) and urea: 94. During evaluation by the ED provider, the patient was noted to have transient slurring of speech, speech arrest, and tonic-clonic movements on the right. CT of the head, followed by CT angiography of the head and neck, demonstrated no acute intracranial pathology. Spot EEG revealed generalized slowing with unclear left-sided epileptiform discharges. There was a concern for complex partial seizures. Neurology and nephrology were consulted. The patient was given 1 g of levetiracetam, and emergent dialysis was performed. After dialysis, no other epileptiform activity was noted with the improvement of her encephalopathy. The patient returned to her baseline mentation. Here we emphasize the importance of recognizing cefepime’s toxicity profile while triaging patients. In the rare event of toxicity, immediate treatment is discontinuing the offending agent and initiation of emergent hemodialysis.

## Introduction

Cefepime is a 4th generation cephalosporin used for its ability to cover gram-positives, gram negatives, anaerobic bacteria, and, most importantly, Pseudomonas aeruginosa. Prior to initiation of cefepime, the Food and Drug Administration (FDA) recommends the medication to be dosed based on the patients' estimated creatinine clearance (CrCl) to avoid a multitude of its toxicity profiles [1]. Severe neurological reactions have been reported, including encephalopathy, aphasia, myoclonus, seizures, and nonconvulsive status epilepticus. These adverse reactions are often seen in patients older than 65 years in the presence of renal impairment with inappropriate renal dosing [2]. This adverse reaction often presents itself in the critical care setting, and rarely appears in the ambulatory setting on arrival to the emergency department (ED), as in our case.

## Case presentation

We present a case of a 65-year-old woman who had developed pseudomonas bacteremia after a complicated femoral artery thrombosis status post transcatheter repair of her mitral valve. The patient was discharged home on a six-week course of IV cefepime for concerns of possible mitral valve endocarditis. Within two weeks of discharge, the patient had been brought back to the ED by her family, with concerns of altered mentation and decreased oral intake. In the ED, the patient had noted to have creatinine: 5.77 (baseline of 0.76) and urea: 94. There was transient slurring of speech, with speech arrest and what appeared to be tonic-clonic movements on the right. On arrival, emergent stroke evaluation was begun. CT of the head followed by CT angiography of the head and neck demonstrated no acute intracranial pathology. Neurology had requested a spot EEG that revealed generalized slowing with unclear left-sided epileptiform discharges. These discharges were concerning for partial seizures. The patient was given 1 g of levetiracetam. Nephrology was consulted for management of acute renal injury leading to cefepime toxicity and emergent dialysis was performed. After dialysis, no other epileptiform activity was noted, with the eventual improvement of her encephalopathy back to her baseline mentation.

## Discussion

Cefepime’s extended spectrum of activity makes it ideal for empiric management of unspecified nosocomial infections. The incidence of cefepime induced neurotoxicity and seizures is unknown and is likely under-reported in the hospital and the ambulatory setting. The elimination of cefepime is principally via renal excretion with an average (±SD) half-life of 2 (±0.3) hours in healthy volunteers. Due to this, it is strongly recommended to adjust the dosage of cefepime in patients with CrCl less than or equal to 60 mL/min [3]. In the setting of outpatient IV cefepime infusions, regular and frequent monitoring of renal function is completed to avoid its toxicities.

The mechanism of myoclonus is not fully understood; the most widely accepted hypothesis is related to concentration-dependent ϒ-aminobutyric acid (GABA) antagonism [1]. Higher concentrations of cefepime in the serum due to decreased clearance by the kidneys, lead to increased blood-brain barrier (BBB) penetration [4]. These risks are often amplified in the critically ill, whereby systemic inflammation disrupts the BBB facilitating further absorption into the brain [5]. The latency of these clinical changes varies significantly in published literature but often neurological deterioration was within five days of initiation of cefepime with renal impairment [6].

A meta-analysis conducted by Appa et al. collected data from 71 separate studies with 198 subjects between January 2013 and June 2016 [7]. The most common clinical manifestations described of toxicity: diminished level of consciousness (80%), agitation (47%), myoclonus (40%), non-convulsive status epilepticus (31%), seizures (11%), aphasia (9%). They noted two dominant EEG patterns: (1) triphasic waves consistent with a toxic metabolic encephalopathy and (2) epileptiform discharges.

A similar metanalysis by Payne et al. analyzing 37 citations with a total of 135 patients had determined predisposing risk factors for neurotoxicity including older age, renal dysfunction, critical illness, history of altered BBB, and elevated unbound drug concentration [8]. The analysis had also reported around one-quarter of patients presenting with neurotoxicity had been dosed appropriately based on CrCl. Suggesting that in the setting of appropriate renal dosing, neurotoxicity and seizures are still a possibility. Unlike the cohorts observed, our patient did not develop neurotoxicity symptoms while inpatient but in the ambulatory setting, ultimately presenting to the ED.

While stroke should always be ruled out in such cases, we also emphasize the importance of recognizing cefepime’s toxicity profile while triaging patients. In the rare event of toxicity, immediate treatment is the discontinuation of the offending agent and initiation of emergent hemodialysis [8]. Antiepileptic and sedative medications have not shown to hasten clinical improvement [9,10].

## Conclusions

With the increasing prevalence of hospital-acquired infections, outpatient antibiotic infusions and the abundant use of cefepime, it is essential for physicians to recognize cefepime's toxicity profile, since early and appropriate intervention is imperative for survival. Whereby changes in mental status typically appear first, myoclonus, aphasia and seizures may develop with continued infusion. And despite appropriate renal dosing of cefepime, toxicity has been reported.
